# SoK: Benchmarking the Performance of a Quantum Computer

**DOI:** 10.3390/e24101467

**Published:** 2022-10-14

**Authors:** Junchao Wang, Guoping Guo, Zheng Shan

**Affiliations:** 1 State Key Laboratory of Mathematical Engineering and Advanced Computing, Zhengzhou 450002, China; 2 CAS Key Laboratory of Quantum Information, University of Science and Technology of China, Hefei 230026, China

**Keywords:** quantum computing, quantum benchmark, fidelity, qubit, quantum circuit

## Abstract

The quantum computer has been claimed to show more quantum advantage than the classical computer in solving some specific problems. Many companies and research institutes try to develop quantum computers with different physical implementations. Currently, most people only focus on the number of qubits in a quantum computer and consider it as a standard to evaluate the performance of the quantum computer intuitively. However, it is quite misleading in most times, especially for investors or governments. This is because the quantum computer works in a quite different way than classical computers. Thus, quantum benchmarking is of great importance. Currently, many quantum benchmarks are proposed from different aspects. In this paper, we review the existing performance benchmarking protocols, models, and metrics. We classify the benchmarking techniques into three categories: physical benchmarking, aggregative benchmarking, and application-level benchmarking. We also discuss the future trend for quantum computer’s benchmarking and propose setting up the QTOP100.

## 1. Introduction

Nowadays, the traditional chip fabrication technique is approaching its limit from 7 nm to even more fine-grained techniques, e.g., 3 nm. When trying to increase the circuit’s density of processor in a limited space, the chip fabrication technique needs to be progressed. However, the current chip fabrication technology almost reaches atom scale. Then, the quantum tunneling effect takes place, leading to the failure of chip fabrication. Thus, researchers have to find new ways to increase the performance of computers. In recent years, with the advancement of the quantum computing technology, quantum computing has become a hot research topic, which causes wide attention. Compared with classical high-performance computers (HPCs), quantum computers have shown advantage in dealing with certain problems. In 2019, a group of researchers from Google first demonstrate quantum advantage, rather than classical HPC, with a Sycamore superconducting processor of 53 qubits. In December 2020, Pan et al. developed a quantum computer (Jiu Zhang) with 76 photons [1]. By executing the task of Gaussian boson sampling (GBS), Pan et al. proved that the JiuZhang is 100 trillion times faster than the Taihu Light supercomputers, which ranked the first of the world’s most powerful computers in 2016 and 2017.

Although quantum computers have great potential in solving certain problems, quantum computers are still in their early stages. When using quantum computers to solve real world problems, there are several problems to be tackled:

(1) Hardware factors. Nowadays, there are different physical implementations for quantum computers, such as trapped ion, superconducting, photon, and semiconducting. Each physical implementation has its own advantages and drawbacks. For instance, the trapped ion quantum computer is relatively easy to control, but the scaling is difficult. In addition, operations on an ion-trap computer take a longer time. The superconducting quantum computer, based on the superconducting Josephson junction, is easy to operate and scalable, but the environmental noise has a great impact on quantum gate (can be seen as quantum instructions) fidelity. Thus, there is currently no perfect physical implementation of a quantum computer. Generally, the quantum circuits (can be seen as quantum programs) executed on quantum processors are prone to error, due to the decoherent quantum property and noise.

(2) Algorithmic factors. The quantum algorithm works in a quite different way from the classical algorithm. It is difficult for people who have been engaged in classical computer algorithm to propose an effective quantum algorithm to solve real world problems. Ideally, the quantum algorithm should use fewer qubits, have shallower quantum circuits and be tolerant of errors. The progress of quantum algorithms was comparatively slow in recent years. Popular quantum algorithms include the prime factor decomposition algorithm (Shor’s algorithm) [2], data search algorithm (Grover algorithm) [3], and quantum approximate optimization algorithm (QAOA) [4].

(3) Application factors. Quantum computers have huge computing potential in solving problems in the areas of finance [5], medicine [6], and artificial intelligence [7]. Many researchers try to use quantum computers to solve real world problems. Nowadays, the solutions cannot work efficiently without the assistance of classical computers. Due to the limitations in hardware and software, most people are pessimistic about quantum computing. They assume that quantum computers will not perform better than the classical computers and are not willing to endeavor adapting their classical implementations to quantum implementations. This limits the wide adoption of quantum computing techniques.

Realizing the problems described above, the number of qubits of a quantum computer cannot represent its computing power. Rather, we need a standardized benchmark to evaluate the power of a quantum computer and what we can do with a quantum computer. In this paper, we review the state of the art of the quantum benchmarking techniques from different aspects and propose a systematic view of these technologies.

The rest of the paper is organized as follows: Section 2 gives a systematic overview of the existing quantum benchmarking technology; Section 3 describes the physical metrics for quantum benchmarking and how these metrics are evaluated; Section 4 reviews the aggregative metrics and benchmarking algorithms; Section 5 summarizes the application-level quantum benchmarking techniques; In Section 6 we discuss the criteria and future trends of quantum benchmarking; Section 7 concludes the paper.

## 2. Overview of Quantum Benchmarks

In this paper, we classify the benchmarks into three categories: the physical benchmarks, the aggregated benchmarks, and application-level benchmarks. Most news and reports place emphasis on the number of qubits in a quantum processor, which is mostly misleading for those who are not familiar with quantum computing. Definitely, the number of qubits can directly decide the quantum computing power of a quantum computer. Some people intuitively think that the quantum computing power of a quantum computer grows exponentially with the number of qubits. For instance, in 2019, Google first demonstrated “quantum supremacy” with a Sycamore quantum processor having 53 qubits. However, apart from the number of qubits, the noise and the quantum property of the qubits can greatly affect the correctness of the results. Thus, apart from the number of qubits, there are other physical properties that most people are concerned about.

**Figure 1:**
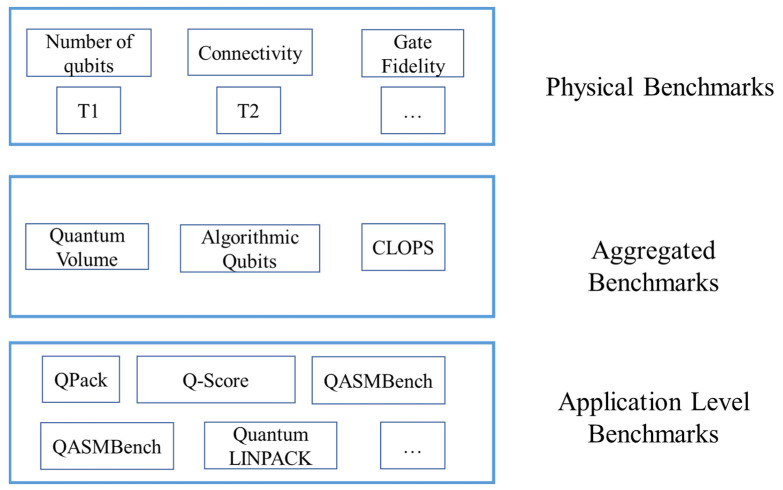
Overview of the quantum benchmarks.

Physical benchmarks include tools, models, and algorithms to reflect the physical properties of a quantum processor. Typical physical indicators of quantum computers include T1, T2, single qubit gate fidelity, two qubit gate fidelity, and readout fidelity. The aggregated benchmarks can help the user to determine the performance of a quantum processor with only one or several parameters. The aggregated metrics can be calculated with randomly generated quantum circuits or estimated based on the basic physical properties of a quantum processor. Typical aggregated benchmarks include quantum volume (QV) and algorithmic qubits (AQ). The application-level benchmarks refer to the metrics obtained by running real-world applications on the quantum computer. Many existing works propose using real world applications to benchmark the quantum computer’s performance because they assume that random circuits cannot reflect a quantum computer’s performance accurately. An overview of the existing quantum benchmarks is shown in Figure 1.

## 3. Physical Benchmarks

Different physical implementations are concerned with different aspects of a quantum computing system. For instance, the trapped ion-based quantum computer focuses more on the stability of the trap frequency, the duration of a gate operation, and the stability of the control lasers. The superconducting quantum computers’ performance is affected by the controllability and scalability of the system. Mostly, they are affected by the precision of the Josephson junction, anharmonicity, and gate duration [8].

In general, the quantum computation systems are concerned with the quantum correlations and controlling operation precision. In a superconducting quantum computer, generally researchers from the background of quantum information focus more on physical properties of quantum computers, such as the T1, T2, number of qubits, connectivity, single qubit gate fidelity, two qubit gate fidelity, and readout fidelity.

The indicators for quantum computers of IBM’s online quantum cloud (Table 1, from [9]) is shown in the following table.

### 3.1. Number of Qubits

In classic computers, “bit” is the basic element for information representation. Each bit has only two states: “1” and “0”. In quantum computers, the quantum operations can be conducted on qubits. Each qubit is a superposition of the “0” state and the “1” state, which can simultaneously represent “0” and “1” with a certain probability. Due to the entanglement between qubits, with *n* qubits, the entangled quantum computer’s superposition state can represent information that needs 2^n^ bits in a classical computer. The more qubits, the more information a quantum computer can describe. Thus, the performance of quantum computers is directly reflected by the number of qubits intuitively [10].

In the early days of the development of quantum computers, the bits in classical computers were relatively stable and simple to operate. However, the quantum properties of qubits are error prone and difficult to maintain. The operations operated on the qubits are extremely complicated. Therefore, designing qubits with high stable quantum properties and operations has become an important problem for quantum processing unit (QPU) designers to overcome.

### 3.2. Qubits’ Connectivity

In a superconducting quantum computer, not all the qubits are directly coupled. Based on the coupling between qubits, we can see the topology of a quantum processor as a graph. The qubits are represented by the vertices, and the edges represent the coupling between qubits. Two qubit gate operation (such as CNOT or Toffolli) cannot be directly applied on two qubits that are not adjacent. This problem can be solved with software solutions by applying SWAP gates, which may extend the length of quantum circuits. Thus, the better the quantum connectivity, the better the performance.

### 3.3. T1 and T2

In quantum mechanics, the energy relaxation time (T1 time) describes the property of a quantum state decaying from a high energy level state |1> approximately to a ground state |0>. The qubit will exchange energy with the external environment, which is referred to as “energy decay” or “energy relaxation”. In a Bloch sphere (each point in the Bloch sphere represents a qubit’s state), the qubit in the excited state is at the south pole, while the ground state is at the north pole. Thus, the T1 describes a longitudinal relaxation rate.

The T1 can be computed with the Rabi experiment. In Rabi oscillation, when the external driving frequency is equal to the frequency of the qubit, the quantum state can flip between two energy levels. Thus, it can be used to measure T1 time. The process includes using a π pulse to excite the quantum state from |0> state to |1> state; when excited to |1> state, after time t, the probability that the qubit is in |1> state is:

(1) $$P\left(|1\rangle \right)=e^{-t/T_{1}}$$

Then, we can use the above formula to get the fitting result of T1 time.

T2* time is another important metric used to benchmark the quality of qubits. T1 focuses on the energy decaying time, while T2* focuses on the pure dephasing time. T2* can be assumed to be a kind of noise that drifts the phase, causing the fluctuations of qubits frequency. Another parameter T2 is a transverse relaxation parameter, combining both the energy relaxation and pure dephasing [11]. Generally, the T1, T2, and T2* have the following relations:

(2) 1T2=12T1+1T2*

Compared with energy relaxation, the pure dephasing is not resonant. Thus, any noise can affect the qubits’ frequency. Since the dephasing is not related to energy exchange with external environment, they can be reduced completely with unitary operations, such as dynamical decoupling pulses [11].

The T2* can be measured with the Ramsey experiment. Ramsey oscillation measures the phase decoherence time. We can apply two *π/2* pulses with an interval of t time for the qubit, and allow the quantum state to freely rotate around the *z* axis in the *x-y* plane within the interval *t*. Then, the phase decoherence time is finally fitted and calculated.

### 3.4. Fidelity

Due to the decoherence of quantum states, a quantum state will change with time. Reduction of gate fidelity is incurred by noise during gate operations. The gate operations can be compared to instructions in a classical computer. A sequence of gate operations applied to a set of qubits can be represented as a quantum circuit (quantum program). Ideally, we can deduce the final state after applying a X gate operation to a qubit. For instance, we can consider the initial state of a qubit as |0>. After applying the X gate, we can obtain the final state of the qubit as |1>. However, due to noise and decoherence, the final state will diverge with the ideal state. The fidelity is used to measure the extent of the divergence between the final ideal state and the real state. There are many sources of uncertain and uncontrollable physical noise in quantum computers. For each quantum gate operation, some errors may be incurred. These errors can also accumulate during the execution of quantum circuit, making the final computing result wrong. Therefore, the quantum computers are error prone during quantum computation. The noise can be introduced from many aspects. One typical type of noise is introduced by external thermal noise. To reduce the noise introduced by heat, the superconducting-based computer should work at an extremely cold temperature, which almost reaches absolute zero. Many researchers try to propose error-correction mechanisms to reduce the error probability. For instance, the “Surface Code” technique tries to build logical qubits based on physical qubits to increase the fidelity and extend the coherence time [12]. Although researchers strive to reduce the error in quantum computers, errors are believed to be an inevitable factor in quantum computing for a long period. Thus, Preskill et al. categorized the quantum computers in different stages, and the quantum computer will remain in the NISQ era in upcoming years [13].

Quantum state tomography (QST) identifies an unknown state after measurement [14]. Quantum process tomography (QPT) and quantum benchmarking techniques can be used to characterize the gate fidelity. With the increase of the number of qubits, the parameters involved in QPT increase dramatically. Thus, QPT is quite time consuming. Other quantum benchmarking technique can be seen as quantum benchmarking protocols, which are different from the quantum benchmarks in this paper. Most quantum benchmarking protocols for estimating gate fidelity are taken from the physical area. For instance, cycle benchmarking protocol is very suitable for large-scale quantum processors [15]. Randomized benchmarking (RB) [16] is used to measure the “average performance” of quantum computer gates. Extended randomized benchmarking (XRB) can be used to estimate the probability of a stochastic error during a random gate.

## 4. Aggregated Benchmarks

Each metric can reflect one aspect of the one or two qubit’s performance. However, the quantum processor consists of many qubits connected with different topology. To better evaluate the performance of a quantum computer, people try to propose an aggregated metric to directly reflect the performance of a quantum processor.

### 4.1. Quantum Volume

When building a larger-scale quantum computer, its performance is susceptible to many factors, such as the number of qubits, connectivity of qubits, and error rate when applying quantum gates. To this end, IBM proposed the quantum volume (QV) [17], a metric used to represent the performance of a quantum computer. Quantum volume is calculated as *2^k^*, where *k* is the largest number of a quantum circuit consisting of *k* qubits and *k*-layer gate operations taken from the Haar-random *SU(4)* unitaries. The QV considers both the number of qubits and the quality of gate operations and measurement. After running the k-layer quantum circuits, the “correct” measured results (heavy output) should be above a certain threshold [18]. Thus, it means that the higher gate fidelity can lead to larger QV.

After the first proposal of quantum volume, many researchers address the flaws of QV. For instance, the quantum circuit used by the quantum volume is a “square”, since it constrains the minimum circuit depth and number of qubits. For some algorithms, the quantum circuit is not “square”. In the factorization algorithm (Shor algorithm), the width of the quantum circuit is *n*, but the depth is *n^3^* [19]. So, QV may not necessarily be the widely accepted quantum benchmarking indicator. Moreover, the QV can be very large in some cases. For instance, the scale of QV reaches almost 4 million for an ion-trap quantum computer [20]. However, for superconducting computers, the QV generally only reaches 128 maximally. This is mainly because the gate fidelity of superconducting quantum computers is below the gate fidelity of the trapped-ion quantum computers. Additionally, the QV is calculated as 2 to the power of the number of high-quality qubits. Thus, the QV can be quite large in trapped-ion quantum computers. Although QV has drawbacks, it is a great endeavor to draw people’s attention to the problems in a quantum computer, instead of only focusing on the number of qubits in a quantum computer [21].

### 4.2. Algorithmic Qubits

The QV metric will become very large because it is exponential to the number of effective width and length for a given circuit. Since the IonQ’s quantum computer has comparatively high gate fidelity, the QV numbers will grow quickly. To address the shortcomings of quantum volume, IonQ introduces a new benchmark—algorithmic qubits. The number of algorithmic qubits (AQ) determines how big a quantum circuit can be executed on a quantum computer. AQ considers error correction and is directly related to the number of qubits. The IonQ’s roadmap for their future quantum computers is based on the AQ metric [22]. IonQ also publishes an online tool for allowing users to calculate the AQ value of a quantum computer, given its basic properties [23].

### 4.3. Mirroring Benchmarks

Proctor et al. concluded that the standard error metrics obtained through random disordered program behavior cannot accurately reflect the performance for some real-world problem [24]. To provide direct insight into a processor’s capability, Proctor et al. built the benchmarks starting from the quantum circuits of varied sizes and structures and transformed the circuits to the mirror circuits that can be efficiently verifiable. In ref. [24], the quantum benchmarks include: the volumetric circuit benchmarks (referring to the IBM quantum volume [19]), the randomized mirror circuits (alternating the layers of randomized Pauli gates and Clifford gates chosen from a sampling distribution), and the periodic mirror circuits (consisting of iteratively germ circuits that can amplify the errors). Proctor et al. assumed that the mirror benchmarks are more efficient, reliable, and scalable for predicting the performance of quantum computing in solving real world problems. The randomized mirror circuit benchmarks are evaluated on twelve processors of IBM quantum computers and Rigetti quantum computers. Their experimental results show that current quantum computing hardware suffers from complex errors. The errors in the structured quantum circuits of real-world applications are quite different from the standard error metrics from random benchmarking techniques. The capability of a quantum processor can be reflected by a similar approach, such as quantum volume. The results also imply that whether a quantum circuit can be successfully run on a quantum processor depends on the circuit’s shape and the exact arrangement of the quantum gates.

### 4.4. CLOPS

In [25], Wack et al. identified three key attributes for evaluating the performance of a quantum computer: quality, speed, and scale. The quality is measured by the quantum volume deciding the maximum size of quantum circuit that can be executed. The scale can be represented by the number of qubits. The speed is measured by the circuit layer operations per second (CLOPS). The CLOPS metric considers the interaction between classical computing and quantum computing because real-world applications include both the classical processing and the quantum processing. The CLOPS is defined as the number of QV layers executed per second. The CLOPS benchmark includes 100 parameterized templated circuits. It allows the system taking all optimizations during the data transfer of circuits and results, run-time compilation, latencies in loading control electronics, initialization of control electronics, gate times, measurement times, reset time of qubits, delays between circuits, parameter updates, and results processing.

Limitations of the benchmark: The CLOPS mostly focuses on the quantum computing part. The computation accounts only for the runtime compilation and optimization. Thus, in CLOPS, the classical computation serves as assistance to the quantum computing. Improvement in the performance of the classical part can barely contribute to the improvement of CLOPS. For an extreme case, when all applications are executed in a classical computer, the CLOPS will be zeroed, since no quantum circuits are executed. Moreover, the CLOPS only considers the time for executing an application, but the quality of qubits and gate operations is reflected in other parameters. From the experimental results of [25], we can see that the quantum circuit execution time only takes quite a small proportion (less than 1%) of the total execution time.

## 5. Application-Based Benchmarks

The physical properties of a quantum computer can affect its performance. However, it is difficult to determine whether a quantum computer outperforms another only based on these properties. For instance, a quantum computer “A” has less qubits, but the qubits’ quality of another quantum computer “B” is higher. If a quantum application needs more qubits, then “A” is preferred. If a quantum application requires the qubits’ quality to be higher, then “B” is preferred. Therefore, some researchers propose to evaluate the performance of a quantum computer with a real-world quantum application.

A summary of the application-based quantum benchmarks is shown in Table 2. In Table 2, we can see that most quantum benchmarks consider the typical combinational optimization problems and use variational quantum circuits (VQC) to solve the problem. This is mainly because the combinational optimization problems can be widely used in many real-world scenarios, such as traffic engineering and flight scheduling. Moreover, the variational quantum solutions, such as quantum approximation optimization algorithm (QAOA) and variational quantum eigensolver (VQE) are popular, due to the possibility to obtain a useful result on NISQ devices. Thus, most people believe that, in the NISQ era, the variational quantum solution will remain the most effective solution.

### 5.1. QPack

QPack is a benchmark including three typical combinational optimization problems: Max-Cut, dominating set, and travelling salesman problem (TSP) [26]. Mesman et al. propose solutions to these problems with a hybrid implementation on classical and quantum hardware. Mesman et al. mainly evaluated the following metrics: runtime, best approximation error, success probability, and performance scaling. Since the QAOA-based implementation cannot be solved without the assistance of classical hardware, the runtime is evaluated based on different aspects. It includes the overall runtime of a hybrid implementation, runtime on classical hardware, connection between classical and quantum hardware, preprocessing and routing, and runtime on quantum hardware. The best approximation error and success probability refers to the extent that QAOA can approximate an optimal solution. The performance scaling is determined by whether the success probability decreases as the problem size grows.

### 5.2. Q-Score

Q-Score considers the quantum solution to the variational optimization problems [27]. To evaluate an application’s performance on a QPU, Q-Score requires the user to provide both the QPU hardware and software stack. Q-Score considers two typical combinational problems, including TSP and Max-Cut. In the open-source implementation of Q-Score, they only provide implementation on the Max-Cut job. Users can customize a random Erdos–Renyi graph and use QAOA to solve the problem [27].

### 5.3. F-VQE

Researchers from Cambridge Quantum proposed a filtering variational quantum eigensolver (F-VQE) to efficiently solve the combinational optimization problem with less qubits [28]. The algorithm is tested by solving random weighted Max-Cut problems on the Honeywell H1 quantum computer. The problem size of Max-Cut reached 23, but the number of qubits required by quantum circuits is less than 6.

### 5.4. Variational Quantum Factoring

Zapata tries to investigate what can be done with NISQ devices. Thus, Zapata proposed to benchmark quantum devices with variational quantum factoring (VQF) and fermionic simulation [29,36]. The VQF solves the integer factoring problem with variational quantum solutions, such as VQE and QAOA. The fermionic simulation includes the 1D Fermi–Hubbard model, which is representative of chemistry and materials science problems. Its analytical solution is known and can be easily extended into a 2D structure, which can be converted to a difficult quantum problem. The metrics for the application benchmark is the effective fermionic length of the device, which can reflect the performance of the entire device [37].

### 5.5. Data-Driven Quantum Circuit Learning Algorithm (DDQCL)

Benedetti et al. perceived that NISQ devices can be used for practical applications with hybrid quantum-classical algorithms (e.g., VQE, QAOA) [30]. Thus, Marcello et al. tried to evaluate the performance of NISQ devices with machine learning application and propose a data-driven quantum circuit learning algorithm (DDQCL) to train shallow circuits for generative modeling, approximating an unknown probability distribution from the data. The problem tries to minimize the Kullback–Leibler (KL) divergence between the quantum circuit probability distribution to the target probability distribution. Benedetti et al. used the particle swarm optimization (PSO) to minimize the cost functions. They used the 2^N^ amplitudes of the wave function and built a Born machine in a quantum computer to find the correlations in a dataset. The generative modeling task in the paper considers the quantum circuit depth, gate fidelities, and connectivity of qubits. To characterize the hybrid quantum-classical hardware’s capability, Benedetti et al. proposed the qBAS (bars and stripes) score. The DDQCL can learn a quantum circuit that encodes all the BAS patterns in the wave function of a quantum state. The qBAS is an instantiation of the F1 score that is widely used in the area of information retrieval, considering both the precision (denoted as p) and recall (denoted as r). The BAS is a synthetic data set of images for generative learning models. The qBAS (n,m) is defined as *2pr/(p + r)*. The precision is defined as the percentage of measurements belonging to the BAS (n,m) dataset. The recall represents the capability to reconstruct the patterns. This metric has been evaluated on an ion-trap quantum computer with GHZ (Greenberger–Horne–Zeilinger) states and coherent thermal states preparation.

### 5.6. 3 Application-Motivated Benchmarks

Daniel et al. assumed that quantum computers should be evaluated on practical tasks [31]. They proposed three application-motivated quantum circuits:

- The deep class of the quantum circuit is taken from the state preparation in the VQE (variational quantum eigensolver) algorithm;
- The shallow class of quantum circuits refer to the quantum circuits whose depth increase slowly with the growth of the width (number of qubits). Shallow is inspired by the IQP (instantaneous quantum polytime)-type quantum circuit, which can be used in quantum machine learning in NISQ. The idea of the shallow circuit is to apply Hadamard gate to all the qubits. Then, a random binomial graph is generated with all the qubits as vertices. The CZ gate acts on all the edges in the graph. After this stage, the Hadamard gate is applied to all the qubits. Finally, the qubits are measured on a computational basis.
- The square class is inspired by the quantum volume benchmark. The circuit’s depth grows linearly with the number of qubits. The quantum circuit is generated by applying quantum gates randomly chosen from *SU(4)* for polynomial times of the circuit width.

The above benchmarks can help to quantify the performance of a quantum computing system after executing the above circuits. Daniel et al. chose three metrics that can be computed with classical computers: heavy output generation probability, cross-entropy difference, and l1-norm distance. The HOG (heavy output generation) evaluates the produced bitstrings, after executing a quantum circuit with the highest probability, that are the most likely in the ideal distribution. The cross-entropy benchmarking is related to the average probability between an ideal distribution and real distribution. The l1-norm distance measures the total divergence of the probability distributions to the sample space. In [31], Daniel et al. assumed that the comprehensive benchmark of the computational capability of a quantum computing system should consider all the aspects that affect the performance: qubits, compilation strategy, and classical control hardware.

### 5.7. Application-Oriented Performance Benchmarks

Due to the complex errors in quantum hardware, a single metric cannot accurately show the performance of the hardware on all applications. For the wide and shallow quantum circuits or deep and narrow circuits, the quantum volume cannot precisely reflect the performance. The component-level performance metrics (physical metrics, such as T1 and T2) are difficult for non-specialists to understand. Thus, Thomas et al. proposed an open-source suite of quantum application-oriented performance benchmarks, including various algorithms and small applications [32]. With their benchmarks, the hardware developers can quantify their progress in quantum hardware. The users can predict the performance of a meaningful computational application on the hardware. The benchmark can benchmark the quality and execution time of a quantum processor.

The selected algorithms and applications include: shallow simple Oracle-based algorithms, quantum Fourier transform (QFT), Grover’s search algorithm, phase and amplitude estimation, Monte Carlo sampling, variational quantum eigensolver (VQE), and Shor’s order finding. Thomas et al. categorized the benchmarks into three classes: tutorial, subroutine, and functional. The “tutorial” class refers to the basic and simple algorithms, e.g., Deutsch–Jozsa, Bernstein–Vazirani, and hidden shift. The “subroutine” class includes the commonly used quantum circuits that work as a critical part of an application. The “functional” class consists of several almost complete applications. In [32], Thomas et al. mainly focused on the quantum computing part. For each benchmark, they generated a set of quantum circuits for different problem sizes. The fidelity for each quantum circuit should be above *1/2*, which corresponds to the heavy output probability of *2/3* in quantum volume. After executing the quantum circuits, Thomas et al. applied the volumetric benchmarking mechanism in quantum volume and evaluated the quality of each benchmark on different problem size and circuit’s depth. The fidelity of an output of a benchmarking case is based on the classical Hellinger distance.

These benchmarks are evaluated on several real quantum computing devices, including Rigetti computing delivered by Amazon Braket Service, IBM Quantum Services, Honeywell System Model H1, and IonQ via Amazon Braket. The benchmark now is open-source and publicly available at [38]. To allow users easily execute on different quantum hardware, the benchmark is implemented with different programming languages, including Qiskit, Cirq, Braket, and Q#. In [32], Thomas et al. improved the quantum volume by introducing three types of benchmarks. However, the benchmark needs many manually pre-set parameters, such as the range of the problem size. The parameters depend on user’s experience. For iterating a wide range of benchmarks, it can be quite slow. Moreover, after benchmarking, although the visualization of the results follows a similar approach as quantum volume, it is still difficult for the users to interpret the visualized results and predict the performance of the hardware on their own quantum hardware. Another important issue has been mentioned in the paper. The proposed benchmark mainly focuses on the quantum computing part. Although Thomas et al. measured the compilation time, the classical computation time, and the quantum execution time, they could not accurately predict the performance of a hybrid quantum application because the capability of a quantum computer is related to the how the quantum system is used in a hybrid application.

### 5.8. Quantum LINPACK

To evaluate the performance of an HPC, Jack Dongard proposed the LINPACK (Linear system package). LINPACK benchmark is used to measure the peak performance of an HPC and appended to the Linpack User’s Guide as an appendix. The benchmark reports the performance for solving a general dense matrix problem (Ax = b) [39]. The program size includes the 100 × 100 problem (can use inner loop optimization), 1000 × 1000 problem (three loop optimization), and a scalable parallel problem (manufacturer can choose the algorithm based on their available memory on their computers). It is based on a mathematical library called the BLAS (basic linear algebra subgprograms).

Inspired by the LINPACK benchmark in classical HPC, Dong et al. proposed a quantum LINPACK benchmark and proposed a random circuit block-encoded matrix (RACBEM), which generalizes a dense random matrix in a quantum problem [33]. The benchmark can be difficult for a classical computer to solve, but the quantum computer can solve it effectively. Thus, it can be used to demonstrate quantum advantage. Dong et al. implemented RACBEM to solve various numerical quantum linear algebra tasks on IBM Q quantum devices and QVMs configured with noise. However, to achieve quantum advantage, the RACBEM requires the high quality of NISQ devices and QRAMs.

### 5.9. Quantum Chemistry

Ref. [34] proposes a quantum chemistry benchmark with a series of electronic structure calculation instances. The quantum algorithmic primitive includes the reduced unitary coupled cluster ansatz (UCC, a state preparation circuit) and hardware-efficient ansatz (variational quantum eigensolver, VQE). The variation of UCC is used to measure the performance and accuracy of quantum computations. The accuracy of VQE is evaluated by recovering the ground state energy for a set of molecules (alkali metal hydrides, NaH, KH, and RbH). The circuit depth of UCC is larger than the HWE ansatz. They evaluated the benchmark on IBM Tokyo and Rigetti Aspen. The benchmark now is open source [40].

### 5.10. QASMBenchmark

QASMBench is a low-level benchmark based on the quantum assembly language OpenQASM [35]. The quantum circuits in the benchmarks are taken from a wide range of applications: chemistry, simulation, linear algebra, searching, optimization, arithmetic, machine learning, fault tolerance, and cryptography. Based on these quantum routines and kernels, Li et al. evaluated the quantum circuit width, depth, gate density, retention lifespan, measurement density, and entanglement variance. The circuit width is directly defined by the number of qubits used in a quantum circuit. The circuit depth is computed as the total time-steps for conducting all the gate operations in a quantum circuit. The gate density considers a quantum circuit as a rectangle, which is denoted as the occupancy of gate operations in the rectangle. The retention lifespan measures the maximum lifespan of qubits, which is closely related to the T1 and T2 coherence time of all qubits. The measurement density evaluates the importance of the measurements. Since the 2-qubit gate heavily affects the error of a quantum circuit, the entanglement variance assesses the balance of entanglement of the qubits in a quantum circuit.

The QASMBench is now open source and available through: [41].

The QASMBench is categorized into three classes, according to the number of qubits used: small scale, with qubits from 2 to 5; medium scale, from 6 to 15; large scale, with more than 15 qubits.

The QASMBench can be converted to other representations, such as Q#, PyQuil, and Cirq, with a q-convert tool [42].

### 5.11. Quantum Supremacy Benchmarking

In recent years, the quantum supremacy or quantum advantage attracts wide attention. The concept of quantum supremacy was first proposed by Preskill in 2012 [43]. The evaluation of quantum supremacy requires the completion of the following four aspects of work:

(1) Define a specific computing problem;

(2) Propose an appropriate quantum algorithm for problem;

(3) Compare the quantum solution with the best result, given by the optimal classical algorithm;

(4) By analyzing the complexity of the quantum algorithm, it is verified that the quantum algorithm can achieve speedup, compared to the classical algorithms.

In [44], Torels et al. defined five different types of quantum speedup. The provable quantum speedup refers to the theoretical proof that no classical algorithm can have better performance than a given quantum algorithm. A strong quantum speedup means the optimal classical algorithm has been found and cannot compete with the quantum algorithm in solving the same problem. The potential quantum speedup refers to comparing with a specific classical algorithm or a set of classical algorithms. The limited quantum speedup refers to comparing the classical algorithm implemented in a same algorithmic approach. A typical example of limited quantum speedup is quantum annealing vs. classical simulated annealing. Usually, the quantum speed-up refers to comparing the best available classical algorithm, but not the best possible classical algorithm [44].

At present, there are several widely used quantum benchmarks to demonstrate quantum advantage, including Shor’s algorithm, random quantum circuit sampling, and boson sampling problem. We briefly introduce the benchmarks and discuss their advantages and limitations.

Shor’s algorithm: The Shor’s algorithm is a polynomial-time quantum algorithm to solve the problem of prime factor decomposition. The prime factor decomposition is one of the most important problems that forms the basis of the modern encryption system. Moreover, the problem is difficult to solve, even with a classical high-performance computer. In [45], Peter Shor first proposed the Shor’s algorithm, which can help to solve the prime factor decomposition problem effectively with a quantum computer. Since then, increasing interest has been attracted by quantum computing. Many companies (e.g., IBM and Google) and research institutes pay a lot of effort to designing and implementing their quantum computers. Intuitively Shor’s algorithm can help demonstrate quantum supremacy. However, to execute Shor’s algorithm in a real quantum computer requires a lot of effort. Craig and Martin combined several techniques together and made a set of plausible assumptions: a quantum processor of a planar grid topology with nearest-neighbor connectivity, a physical gate error rate of 10^−3^, a surface code cycle time of 1 microsecond, and a reaction time of 10 microseconds. Gidney et al. estimated that factoring 2048-bit RSA integers may need 20 million qubits [46]. The above analysis is based on many underlying assumptions, and Jinyong et al. further analyzed the required quantum resources of implementing Shor’s algorithm under different conditions. Their work is also based on some assumptions that quantum error correction (rotated surface code) and all-to-all connectivity of logical qubits are used. Shor’s algorithm depends on the modular exponentiation. Beauregard algorithm uses a smaller number of logical qubits. Pavlidis algorithm may require less depth when logical qubits are used. In a quantum computer with 2048 qubits and error rate of gate operation of 10^−3^, the Beauregard algorithm requires 12 million physical qubits for data and almost 1 million physical qubits for magic-state factories [47]. The Pavlidis algorithm requires almost 50 million physical qubits for data and almost 600 million physical qubits for magic-state factories [48,49].

From the above description, we can see that, although Shor’s algorithm can accelerate the factoring of integers theoretically, running the Shor’s algorithm in a real quantum computer is non-trivial. Thus, the current quantum computers are far from demonstrating the Shor’s algorithm and show quantum advantage.

Random circuit sampling: Random circuit sampling (RCS) [50] refers to sampling from the probability distribution of randomly selected quantum circuits. In June 2021, a team from the University of Science and Technology of China developed a 66-qubit superconducting processor “Zuchongzhi” and run random line sampling [51], which is expected to be 2–3 orders of magnitude higher than Google’s 53-qubit “Sycamore” quantum processor.

Boson Sampling [52] refers to sampling the probability distribution of sent bosons through a linear optical network. It is a computational problem that quantum computers can solve more effectively than classical computers. Thus, it can be one of the candidates for evaluating quantum supremacy. It is mentioned in the introduction that 76 photon quantum computing prototypes “Jiuzhang” have realized the calculation of “Gaussian Boson Sampling (GBS)”, and its calculation speed is 100 trillion times that of supercomputers. However, the random quantum circuit sampling problem cannot be directly used for solving real world problems. Moreover, as the scale of the problem increases, the depth of the quantum circuit will increase dramatically. Many experts from the high-performance computing area improves the performance of quantum simulators by optimizing the quantum simulation algorithm with the HPC architecture, which can also accelerates the execution of RCS and Boson sampling problems in a high-performance computer [53,54].

Moreover, the sampling problem cannot be applied in real-world scenarios directly. Thus, most researchers argue with sampling problems, and the quantum computer remains to be a physical device that is not effective in solving real world problems.

## 6. Discussion

In recent years, many countries have elevated the development of quantum computers to a national strategic level. More and more quantum computers are designed and implemented. To allow users to easily identify the computation capabilities of the quantum computers, instead of only focusing on the number of qubits, the quantum benchmarking becomes increasingly important. Apart from the metrics mentioned above, there are other aspects we need to consider:

(1) Stability of quantum computers.

Although we can calculate the average gate fidelity through benchmarking protocol such as RB, the gate fidelity can vary with time. When executing quantum programs, the gate fidelity is not always trustworthy. For experimental researchers working on quantum computing, they are putting a lot of effort into improving the optimal gate fidelity and coherence time. The stability is mainly affected by the hardware sources of noise (e.g., flux noise and oscillator drift) and how frequently and accurately calibrations are repeated.

Based on our observation, the QV is also not stable, due to the variance of gate fidelity and coherence time. Usually, the claimed QV is measured at the best performance. However, when a user uses a quantum computer, the QV may not reach the claimed number. The TOP500 is also concerned with the peak performance of the HPCs. However, the quantum computer works in a quite different way. Although the peak performance of an HPC can vary, the variance of the performance of a quantum computer is quite large.

To the best of our knowledge, there is no benchmarking criteria considering the stability of the quantum computer’s performance directly.

(2) Criteria for building a quantum benchmark.

Although there is no universal quantum benchmark for quantum computers, we summarize the criteria for benchmarking a quantum computer:

- The benchmark should be representative of real-world problems. In the early stages of SPEC, people mostly use HPC to solve linear equations. Nowadays, AI is becoming a hot topic. Thus, the benchmarking approach of SPEC includes more AI solutions.
- The benchmark should demonstrate quantum advantage. Nowadays, theoretic quantum researchers try to find effective quantum solutions to real world problems and problems that can help to demonstrate quantum advantage. For instance, Rooya Ronagh proposed a quantum algorithm for solving finite-horizon dynamic programming problems [55]. Some sampling problems can be easily solved in a quantum computer, but for a classical computer, the problem is not so easy. Some people may argue the quantum computer is a physical instrument that cannot be used for computing. Therefore, we highlight using quantum computer to solve real world problems.
- The benchmark should be easily customizable. For instance, the test set should not only require the number of qubits to exceed a certain threshold. For a quantum computer with high gate fidelity, but less qubits, the test set should also work.

Although the benchmark for classical supercomputers has been set up for almost 30 years, the benchmark evolves gradually with the development of human needs and HPCs. In recent years, people focus on the power consumption of an HPC. Thus, Green 500 is used to measure the energy efficiency of an HPC. It is evaluated as the performance per watt [56]. GRAPH500 is a benchmark proposed to evaluate the performance of supercomputers in solving the large graph problems [57].

(3) Benchmarking in a heterogeneous systems

With the development of quantum computing technology, more and more quantum processors are produced. Due to the different physical implementations of quantum computers (superconducting, trapped ion, photon, nuclear magnetic resonance (NMR), quantum dots) and different properties of quantum processors (e.g., processor topology, T1, T2, and gate fidelity), the quantum processors can be connected as a distributed heterogenous system, similar to how a hybrid classical computing cluster consisted of heterogenous computing nodes. A hybrid classical computing cluster consists of nodes with different number of CPUs, GPUs, and FPGAs. The CPU computing nodes may also be heterogenous of different number of CPU cores and memories. The quantum processors can facilitate the distributed quantum computing.

In the future, it is essential to take advantage of different quantum processors. For a real-world application, it may include modules that need quantum processors with shallow depth, but more qubits, as well as quantum processors with deeper depth, but less qubits.

(4) Set up the QTOP100 list of quantum computers.

The TOP500 list was created in 1993 to rank the 500 world’s fastest computer [56]. Twice every year, the committee of TOP500 updates the TOP500, based on the running performance of HPL N*N on these computers. We can refer to the success of TOP500, and set up the QTOP100 in the quantum computing area.

## 7. Conclusions

By comparing the benchmark technology of high-performance computers, this paper addresses the importance of quantum benchmarks and summarizes the existing technologies on how to benchmark the performance of a quantum computer. We classify the existing technologies into three categories: physical level benchmark, aggregated level benchmark, and application-level benchmark. We can see that a perfect quantum benchmark does not exist.

We also discuss the future trends of quantum benchmarking. The quantum benchmarking techniques will change with time, similar to the benchmarks in the HPC area. In the future, we believe that the quantum benchmarks will be more concerned with the real-world problems. The stability and robustness of a quantum computer will be heavily addressed.

## Figures and Tables

**Table 1:** IBM quantum cloud’s performance metrics. Avg stands for average; N/A means not applicable.

Name	Number of Qubits	QV	Avg.T1 (μs)	Avg.T2 (μs)	Avg.Readout Fidelity	Avg.CNOT Fidelity
brooklyn	65	32	77.1686	74.6345	0.9682	0.9746
manhattan	65	32	110.1959	101.6078	0.9761	0.9543
hanoi	27	64	123.3959	93.4341	0.9837	0.991
sydney	27	32	266.1433	256.6081	0.9833	0.9898
peekskill	27	N/A	97.4474	107.0911	0.9821	0.9896
cairo	27	64	76.01	97.6543	0.9796	0.989
toronto	27	32	180.3614	155.1329	0.9869	0.9814
kolkata	27	128	70.3363	75.2432	0.9698	0.9536
mumbai	27	128	117.2574	92.1067	0.9484	0.9526
montreal	27	128	81.004	104.678	0.938	0.4972
guadalupe	16	32	132.6257	40.5357	0.977	0.9896
lagos	7	32	158.6	57.702	0.9697	0.9912
jakarta	7	16	74.214	104.008	0.9728	0.9895
perth	7	32	155.0078	92.217	0.9118	0.9894
casablanca	7	32	82.2681	96.0744	0.9696	0.9883
nairobi	7	32	86.5337	107.1733	0.9428	0.9878
quito	5	16	130.2629	100.9629	0.9859	0.9932
santiago	5	32	105.2286	98.9143	0.9633	0.9909
manila	5	32	100.56	101.29	0.9739	0.99
lima	5	8	84.0278	84.4122	0.9829	0.9891
belem	5	16	75.936	94.722	0.9676	0.9828
bogota	5	32	92.454	124.096	0.959	0.9794
armonk	1	1	118.1	149.22	0.967	N/A

**Table 2:** Summary of the application-based quantum benchmarks.

Reference	Benchmark Name	Problems	Solution	Metrics
[26]	Qpack	Max-Cut, dominating set, and travelling salesman problem (TSP)	VQC	Runtime, best approximation error, success probability, and performance scaling
[27]	Q-Score	TSP and Max-Cut	VQC	Q-Score
[28]	F-VQE	Max-Cut	VQC	N/A
[29]	Variational quantum factoring (VQF) and fermionic simulation	Variational quantum factoring (VQF) and fermionic simulation	VQC	The effective fermionic length of the device
[30]	Machine learning application	Approximating an unknown probability distribution from data	Data-driven quantum circuit learning algorithm (DDQCL).	qBAS (bars and stripes) score
[31]	3 application-motivated quantum circuit	N/A	The quantum circuits include: the deep class of the quantum circuit is taken from the state preparation in the VQE (variational quantum eigensolver) algorithm; the shallow class of quantum circuits refers to the circuits whose depths increases slowly with the growth of width (number of qubits); square is inspired by the quantum volume benchmark.	Heavy output generation probability, cross-entropy difference and l1-norm distance
[32]	Application-oriented performance benchmarks	N/A	The quantum circuits of the benchmark include: shallow simple Oracle-based algorithms, quantum Fourier transform (QFT), Grover’s search algorithm, phase and amplitude estimation, Monte Carlo sampling, variational quantum eigensolver (VQE), and Shor’s order finding.	The quality and execution time
[33]	Quantum LINPACK	Dense random matrix in a quantum problem	RAndom Circuit Block-Encoded Matrix (RACBEM).	N/A
[34]	Quantum chemistry benchmark	Electronic structure calculation instances	reduced unitary coupled cluster ansatz (UCC, a state preparation circuit) and hardware-efficient ansatz (Variational Quantum Eigensolver, VQE).	Performance and accuracy
[35]	QASMBench	N/A	Quantum circuits are taken from chemistry, simulation, linear algebra, searching, optimization, arithmetic, machine learning, fault tolerance, cryptography.	circuit width, depth, gate density, retention lifespan, measurement density and entanglement variance

We summarize the existing application-level quantum benchmarks as follows.
